# Hemodynamics and tissue oxygenation effects after increased in positive end-expiratory pressure in coronary artery bypass surgery

**DOI:** 10.1186/s40945-016-0030-4

**Published:** 2017-01-11

**Authors:** Vanessa Marques Ferreira Méndez, Mayron F. Oliveira, Adriana do Nascimento Baião, Patrícia Andrade Xavier, Carlos Gun, Priscila A. Sperandio, Iracema I. K. Umeda

**Affiliations:** 1 Physiotherapy Unit, Dante Pazzanese Institute of Cardiology, Avenida Dr Dante Pazzanese, 500, CEP: 04012-180, Vila Mariana, São Paulo, SP Brazil; 2 Physiotherapy Unit, Division of anesthesiology and intensive care unit – Federal University of São Paulo (UNIFESP), São Paulo, SP Brazil; 3 Physiotherapy Unit, Division of Health Sciences Centre, University of Fortaleza (UNIFOR), Fortaleza, CE Brazil; 4 Physiotherapist of Hospital Israelita Albert Einstein (HIAE), São Paulo, SP Brazil; 5 Medical doctor of Dante Pazzanese Institute of Cardiology – Division of Intensive Care Unit, São Paulo, SP Brazil

**Keywords:** PEEP, ScvO_2_, Cardiac surgery, Hemodynamics, Physiotherapy

## Abstract

**Background:**

Cardiac surgery is widely used in the treatment of cardiovascular diseases. However, several complications can be observed during the postoperative period. Positive end expiratory pressure (PEEP) improves gas exchange, but it might be related to decreased cardiac output and possible impairment of tissue oxygenation. The aim of this study was to investigate the hemodynamic effects and oxygen saturation of central venous blood (ScvO_2_) after increasing PEEP in hypoxemic patients after coronary artery bypass (CAB) surgery.

**Methods:**

Seventy post-cardiac surgery patients (CAB), 61 ± 7 years, without ventricular dysfunction (left ventricular ejection fraction 57 ± 2%), with hypoxemia (PaO_2_/FiO_2_ ratio <200) were enrolled. Heart rate, mean arterial pressure, arterial and venous blood samples were measured at intensive care unit and PEEP was increased to 12 cmH_2_O for 30 min.

**Results:**

As expected, PEEP12 improved arterial oxygenation and PaO_2_/FiO_2_ ratio (*p <* 0.0001). Reduction in ScvO_2_ was observed between PEEP5 (63 ± 2%) and PEEP12 (57 ± 1%; *p* = 0.01) with higher values of blood lactate in PEEP12 (*p <* 0.01). No hemodynamic effects (heart rate, mean arterial pressure, SpO_2_; *p >* 0.05) were related.

**Conclusion:**

Increased PEEP after cardiac surgery decreased ScvO_2_ and increased blood lactate, even with higher O_2_ delivery. PEEP did not interfere in hemodynamics status in CAB patients, suggesting that peripheral parameters must be controlled and measured during procedures involving increased PEEP in post-cardiac surgery patients in the intensive care unit.

## Background

Coronary artery bypass (CAB) surgery is widely used in cardiovascular treatment and, during procedure, patients are exposed to anesthesia and cardiopulmonary bypass. This exposure is one of the most important cause of pulmonary dysfunction in the postoperative period with higher levels of morbidities [1]. Moreover, anesthesia might be responsible for reduced pulmonary volume, atelectasis and reduced oxygenation [1].

In this scenario, positive end expiratory pressure (PEEP) has commonly been used to reverse atelectasis and hypoxemia [2]. However, PEEP could interfere in intra-thoracic pressure, which can reduce venous return and cardiac output [3], which in turn can affect tissue perfusion and delivery/utilization.

The influence of PEEP on cardiac output and tissue oxygenation can be measured by oxygen saturation of central venous blood (ScvO_2_) [3] and values under 70% suggest low oxygen delivery/utilization and are predictive of a poor prognosis during postoperative period [3]. However, there are few studies that evaluated the hemodynamic effects of PEEP and ScvO_2_ in patients after cardiac surgery (CAB). Therefore, the aim of this study was to evaluate hemodynamic effects and tissue oxygenation measured by ScvO_2_ in two levels of PEEP in patients after CAB.

## Methods

Post elective cardiac surgery patients (CAB), over 40 years, with hypoxemia (PaO_2_/FiO_2_ ratio <200) and hemoglobin >10 g/dL were enrolled. Patients with hemodynamic instability (heart rate <50 bpm or >140 bpm), mean arterial pressure <60 mmHg or >140 mmHg, norepinephrine dosage >0.03 mcg/kg/min and left ventricular ejection fraction <40% were excluded.

**Fig. 1:**
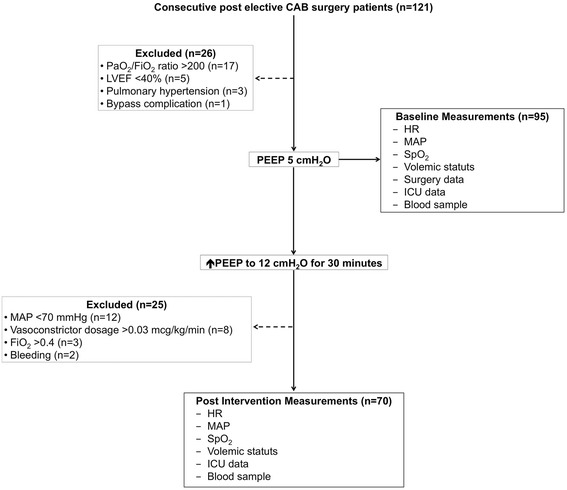
Flowchart of input in the study. Figure notes: LVEF = left ventricular ejection fraction; HR = heart rate; MAP = mean arterial pressure; SpO_2_ = hemoglobin saturation by pulse oximetry; ICU = intensive care unit; FiO_2_ = oxygen support

After undergoing cardiovascular surgery, all of the patients were admitted to the intensive care unit (ICU) and immediately arterial and central venous blood sample were collected and submitted for blood gas analysis (865(R) blood gas analyzer; Chiron Diagnostics, England). The catheter’s for venous blood sample was located at the right atrium and the correct position was checked accordingly to the institutional protocol. Heart rate, mean arterial pressure, and oxyhemoglobin saturation by pulse oximetry (SpO_2_) were measured with a Dixtal monitor (DX 2010®); volemic status (water balance) was also monitored during surgery and into the protocol. All patients were admitted at ICU and followed the institutional mechanical ventilation protocol with volume-controlled (PEEP 5 cmH_2_O, respiratory rate 14 breaths per minute, FiO_2_ 0.4, tidal volume of 6–8 mL/kg) and PEEP was increased to 12 cmH_2_O for 30 min in all patients with arterial hypoxemia (PaO_2_/FiO_2_ ratio <200) (Fig. 1). After 30 min, all variables were measured and the protocol was ended.

Study protocol was interrupted and PEEP was returned to 5 cmH_2_O if there was drop in SpO_2_ below than 90%, increased in FiO_2_, mean arterial pressure <70 mmHg or bleeding >200 mL. All subjects provided written informed consent, and the study was approved by the Medical Ethics Committee of the Dante Pazzanese Institute of Cardiology, São Paulo, Brazil (protocol number 3535).

### Statistical analysis

Statistical analysis was carried out using the SPSS program (version 18.0. Chicago: SPSS Inc.). The Kolmogorov–Smirnov test was used to determine normality of the data distribution. Categorical variables were presented in absolute frequencies and percentage values. Continuous variables of parametric distribution were presented as mean ± standard deviation (SD) and, when appropriated, median [minimum value; maximum value] for variables of non-parametric distribution. Paired *T*-test and Mann–Whitney were used for comparisons between conditions. For all the analyses the statistical significance was accepted when *p <* 0.05.

## Results

One hundred and twenty one consecutive post elective cardiovascular surgery patients were enrolled in this study and none of the patients had pulmonary disease, heart failure or renal insufficiency prior to surgery; fifty-one patients were excluded (bypass complication; LVEF <0.4; pulmonary hypertension; bleeding; MAP <70 mmHg; vasopressor medication >0.03 mcg/kg/min; higher oxygen support – FiO_2_ > 0.4); afterwards seventy patients were included in the study (Fig. 1).

Baseline characteristics are shown in Table 1. There were a higher percentage of male patients (77%) (Table 1) and, during surgery procedure, 50 patients (71%) had positive volemic status, and it remained positive during the stay in the intensive care unit, at both PEEP 5 cmH_2_O (180 ± 82 mL) and PEEP 12 cmH_2_O (197 ± 78 mL) (*p >* 0.05). It should be noted that during the protocol all patients had 0.4 of oxygen support.

**Table 1:** Patient’s characteristics and surgical data in seventy patients after CAB surgery

*Anthropometrics/Demographics*	
Age, years	61 ± 7
Weight, kg	73 ± 10
Height, m	1.63 ± 0.11
BMI, kg/m^2^	27 ± 4
LVEF, %	57 ± 2
EuroSCORE, median (min-max)	5 (3–9)
ICU, days	2 ± 1
Mechanical ventilation time, hrs	6 ± 2
Hospital length of stay, days	7 ± 2
*Main comorbidities*	
Hypertension, n (%)	64 (91%)
Dyslipidemia, n (%)	52 (74%)
Diabetes, n (%)	40 (57%)
Ex-smoker, n (%)	35 (50%)
*Surgical data*	
Partial thromboplastin time activated, s	19 ± 8
Cardiopulmonary bypass time, min	107 ± 22
Cross-clamp time, min	61 ± 17

Abbreviations: *BMI* body mass index, *LVEF* left ventricular ejection fraction, *EuroSCORE* European System for Cardiac Operative Risk Evaluation. Data are expressed as mean values ± standard deviation, unless specified

As expected, 12 cmH_2_O of PEEP increased PaO_2_ and improved PaO_2_/FiO_2_ ratio (Table 2). Moreover, no differences in carbon dioxide pressure (PaCO_2_) were observed between the two interventions. On the other hand, increased PEEP significantly reduced tissue oxygenation observed by ScvO_2_ with higher values of blood lactate but without difference in MAP or in central venous pressure (Table 2).

**Table 2:** Hemodynamic and tissue oxygenation in seventy patients after CAB surgery

	PEEP 5	PEEP 12	*p*-value
HR, bpm	97 ± 16	92 ± 14	0.02
SAP, mmHg	109 ± 13	122 ± 17	0.12
DAP, mmHg	77 ± 16	81 ± 12	0.58
MAP, mmHg	89 ± 15	93 ± 13	0.65
Double product	10593 ± 411	11214 ± 459	0.17
Central venous pressure, mmHg	7 ± 2	8 ± 3	0.96
SpO_2_, %	94 ± 3	96 ± 3	0.89
V_T_, mL	597 ± 121	613 ± 130	0.33
RR, ripm	14 ± 1	14 ± 2	0.99
Catecholamine	12 (17%)	15 (21%)	0.21
*Blood sample*			
Hb, g/dL	12 ± 1	12 ± 2	0.92
pH	7.37 ± 0.07	7.35 ± 0.06	0.05
PaO_2_, mmHg	70.2 ± 6.2	112.8 ± 2.1	<0.0001
PaCO_2_, mmHg	38.6 ± 8.8	38.7 ± 7.8	0.96
HCO_3_^−^	22.1 ± 2.3	21.8 ± 1.6	0.03
BE	−2.4 ± 0.8	−3.5 ± 0.6	0.04
SaO_2_, %	93 ± 3	97 ± 2	0.02
PaO_2_/FiO_2_ ratio	179 ± 24	271 ± 38	<0.0001
ScvO_2_, %	63 ± 5	57 ± 3	0.01
Lactate, mmol/L	3.2 ± 1.1	6.7 ± 1.5	0.001

Abbreviations: *HR* heart rate, *bpm* beats per minute, *SAP* systolic arterial pressure, *DAP* diastolic arterial pressure, *MAP* mean arterial pressure, *SpO*_2_ hemoglobin saturation by pulse oximetry, *V*_*T*_ tidal volume, *RR* respiratory rate, *ripm* respiratory incursion per minute, *Hb* hemoglobin, *pH* hydrogen potential, *PaO*_2_ arterial oxygen pressure, *PaCO*_2_ arterial carbon dioxide pressure, *HCO*_3_^−^ bicarbonate, *BE* base excess, *SaO*_2_ arterial oxygen saturation, *ScvO*_2_ oxygen saturation of central venous blood. Values are expressed in mean ± standard deviation or frequency

Most patients were discharged from the intensive care unit on the second day and with low mechanical ventilation time (Table 1). None of the patients included in the study experienced cardiac outcome or death after the protocol.

## Discussion

In the present study we investigated the influence of PEEP on hemodynamics and tissue oxygenation in patients after CAB. It should be noted that increases in PEEP caused a significant reduction in tissue oxygenation (ScvO_2_), even with higher levels of oxygen delivery (PaO_2_ and PaO_2_/FiO_2_ ratio).

Collapsed lung and hypoxia are common complications after cardiac surgery, mainly due to anesthesia (decreased muscle tone and predisposition to atelectasis) [4] and these changes may be accentuated by surfactant reduction and inflammatory response. In addition, the inflammatory process contributes to pulmonary interstitial edema, which cause a significant decrease in pulmonary gas exchange [5]. Our results indicate increases in PaO_2_, oxygen hemoglobin saturation, and PaO_2_/FiO_2_ ratio, suggesting a reversal of alveolar collapse. These results are consistent with those found in the scientific literature, mainly in patients with acute respiratory distress syndrome [5]. Studies have shown that higher values of PEEP can prevent hypoxemia, with a significant increase in PaO_2_ in post-cardiac surgery patients [5–7].

Despite the benefits of high PEEP after cardiac surgery in improving oxygenation, some precautions should be taken with PEEP increases in patients at the intensive care unit, mainly because PEEP might interfere with intra-thoracic pressure with hemodynamic effects. Some studies have demonstrated that reductions in cardiac output and tissue oxygenation could occur after increased PEEP [4, 8]. Moreover, different PEEP levels reduced cardiac output without changes in PaO_2_/FiO_2_ ratio, ScvO_2_, or hemodynamic values (heart rate or mean arterial pressure). The authors suggested that the reduced cardiac output was due to high levels of PEEP, as cardiac output was restored after PEEP decreased to 5 cmH_2_O [8].

Studies of normovolemic patients have reported maintenance of cardiac output after increased PEEP [9]. However, it should be noted that our study exhibited positive water balance and higher values of blood lactate after study protocol, suggesting that increased PEEP values were responsible for reducing cardiac output and, consequently, for reductions in tissue oxygenation (lower ScvO_2_ values). Recently, Gutierrez (2016) [10] described a higher respiratory muscle workload with a decreased ScvO_2_ and this finding was not associated with sepsis. Furthermore, Rivers et. al. (2001) [11] demonstrated that increases in ScvO_2_ should have been related to respiratory muscles unloading by mechanical ventilation. Our study was not designed to access the respiratory muscle function (overloading or unloading) by PEEP increases, but this fact might have influenced the reduction in ScvO_2_ values. This issue should be performed in further studies with PEEP hemodynamics after CAB surgery. Moreover, some studies have demonstrated that higher levels of PEEP required more sedatives and increase the mechanical ventilation time and compromise the mechanical ventilation withdrawal [6, 12, 13]. Our data support the notion that high PEEP levels directly affect tissue oxygenation in patients after cardiac surgery, even with preserved cardiac function and without surgery complications. However, it should be noted that our protocol did not measure cardiac output directly and we suggest a reduction in cardiac output using ScvO_2_.

Moreover, some studies have reported that short periods of increased PEEP may be beneficial to post-cardiac surgery patients without compromising hemodynamic or tissue oxygenation [14]. In this scenario, even in short periods of high PEEP levels, certain precautions should be taken, such as ScvO_2_ measurements. Our results suggest a reduction in tissue oxygenation and higher levels of blood lactate during only 30 min of rising PEEP; and it has been demonstrated that reductions in ScvO_2_ are related to poor outcomes after cardiac surgery. The maintaining ScvO_2_ above 70% resulted in a reduction in mortality [15] and our findings support our suggestions that ScvO_2_ should be monitored in patients without cardiac failure or without surgery complications.

### Study limitations

Some limitations of this study should be addressed. The inclusion criteria of PaO_2_/FiO_2_ ratio <200 may have limited the number of patients in our study. Thus the extrapolation of our findings to other studies of CAB surgery should be viewed with caution. On the other hand, there is evidence that the PaO_2_/FiO_2_ ratio <200 is the most common hypoxemia finding after CAB surgery [16] and PaO_2_ alterations in patients after CAB surgery do not depend on PEEP only but the level of O_2_ delivery/utilization ratio [11]. Moreover, we did not measure cardiac output before or after PEEP increase, which could have better demonstrated tissue oxygen delivery and utilization. Rather, ScvO_2_ values were used to reflect tissue oxygen delivery and utilization indirectly during the PEEP maneuver. The venous blood sample has been taken by a catheter positioned in right atrium, we recognize that this could rise some questions, but all patients had normal right ventriculum and with adequate volemic status and central venous pressure. Further studies with Swan-Ganz catheter should be performed to access the directly effect of hemodynamics repercussions with PEEP in hypoxemic CAB patients. We believe that ScvO_2_ is a good alternative to measure whole-body oxygen extraction, it is minimally invasive and have low cost. In addition, our results demonstrated positive water balance during the intensive care unit, but other measurements could also have been addressed, such as Swan-ganz catheter. Moreover, we did not measure the inflammation status after cardiac surgery or after PEEP maneuver.

Our study aimed to evaluate the hemodynamics effects after PEEP increases, and we recognize that a control group, without PEEP increases, was not performed; but the PEEP titration was not main objective of the study; we aimed to investigate hemodynamics and tissue oxygenation after a simple PEEP increase in hypoxemic patients after CAB. The choice of 12 cmH_2_O for the PEEP value was made because it is commonly used in intensive care units to reverse arterial hypoxemia [17]. Further studies for hemodynamics measurements and tissue perfusion with control group and with different groups of PEEP are necessary, both in CAB and other cardiac procedures.

## Conclusion

In this study, increases in PEEP after CAB decreased ScvO_2_ and increased blood lactate, even with higher O_2_ delivery. In addition, PEEP increases did not interfere in hemodynamics status in CAB patients, suggesting that in addition to arterial blood gas analysis and hemodynamics measurements, peripheral parameters must be controlled and measured during procedures involving increased PEEP in post-cardiac surgery patients in the intensive care unit.
